# Metabolomic Analysis of the Effect of Postnatal Hypoxia on the Retina in a Newly Born Piglet Model

**DOI:** 10.1371/journal.pone.0066540

**Published:** 2013-06-18

**Authors:** Rønnaug Solberg, Javier Escobar, Alessandro Arduini, Isabel Torres-Cuevas, Agustín Lahoz, Juan Sastre, Ola Didrik Saugstad, Máximo Vento, Julia Kuligowski, Guillermo Quintás

**Affiliations:** 1 Department of Pediatric Research, Institute for Surgical Research, Oslo University Hospital - Rikshospitalet, Oslo, Norway; 2 Neonatal Research Group, Health Research Institute Hospital La Fe, Valencia, Spain; 3 Department of Physiology, Faculty of Pharmacy, University of Valencia, Valencia, Spain; 4 Hepatología Experimental y Trasplante Hepático, Health Research Institute Hospital La Fe, Valencia, Spain; 5 Division of Neonatology, University & Polytechnic Hospital La Fe, Valencia, Spain; 6 Leitat Technological Center, Bio In Vitro Division, Barcelona, Spain; Imperial College London, United Kingdom

## Abstract

The availability of reliable biomarkers of brain injury secondary to birth asphyxia could substantially improve clinical grading, therapeutic intervention strategies, and prognosis. In this study, changes in the metabolome of retinal tissue caused by profound hypoxia in an established neonatal piglet model were investigated using an ultra performance liquid chromatography – quadrupole time of flight mass spectrometry (UPLC-QTOFMS) untargeted metabolomic approach, which included Partial Least Squares – Discriminant Analysis (PLSDA) multivariate data analysis. The initial identification of a set of discriminant metabolites from UPLC-QTOFMS data was confirmed by target UPLC-MS/MS and allowed the selection of endogenous CDP-choline as a promising candidate biomarker for hypoxia-derived brain damage assessing intensity of retinal hypoxia. Results from this study will foster further research on CDP-choline changes occurring during resuscitation.

## Introduction

Perinatal asphyxia, defined as a severe lack of oxygen and perfusion to the fetus during labor and delivery, is a worldwide problem and a foremost cause of mortality and permanent neurodevelopmental disabilities [1], [2]. Short and long-term prognosis following perinatal asphyxia will closely correlate with the subsequent degree of hypoxic ischemic encephalopathy (HIE) [3]. Interestingly, the use of therapeutic hypothermia has substantially increased survival and improved neurocognitive outcome in babies with moderate HIE. However, it has only been limited when applied to patients with the severe form of HIE [4], [5]. The current approach to diagnosis of birth asphyxia in the delivery room evolving to HIE relies on qualitative clinical evaluation [6]. Once in the neonatal intensive care unit diagnosis will be confirmed using an ample array of diagnostic tools such as amplitude integrated electroencephalography, conventional electroencephalography, brain ultrasound and Doppler, and magnetic resonance imaging [6]. In this scenario, the possibility of an early and accurately grading of brain damage using a reliable biomarker could be extremely useful for clinicians to decide about the most appropriate therapeutic approach, risk stratification and prognosis [7].

A molecular biomarker is defined as a traceable substance that can be objectively measured and evaluated as an indicator of a physiological as well as a pathological process or pharmacological response to a therapeutic intervention [8]. Therefore, specific molecular modifications on DNA, RNA, proteins or metabolite levels can be useful biomarkers. Despite considerable efforts focused on genomics and proteomics, molecular biomarkers found to be useful in the diagnosis of perinatal asphyxia are lacking [9], [10]. The most widely employed markers of brain damage after a hypoxic episode include S100B, neuron-specific enolase, activin A, adrenomedullin, Interleukin (IL)-1β and IL-6. However, elevation of these biomarkers can also be the consequence of a variety of conditions not related to asphyxia or may be released by tissues different from brain [11]. Conspicuously, the therapeutic window between the hypoxic/ischemic insult and the subsequent encephalopathy is relatively short (up to 6–8 h after birth) [11]. Therefore, it would be desirable to have available biomarkers consistent with ongoing pathophysiologic changes in the brain and easy and rapid to determine. In this context, metabolite biomarkers offer several advantages over genes and proteins including easier and faster analytical quantification. Metabolomics has become a rapidly growing area of Systems Biology that reflects the downstream products of gene regulation and expression. Accordingly, it can be considered that the metabolome delivers a dynamic snapshot of the functional level of a biological system more appropriate than those provided either by genomics or proteomics [12], [13]. The increasing use of metabolomics to gain further insight into the medical conditions affecting the neonate and the developing human being [14] as well as in different areas of neonatology has been recently reviewed [15]. In a previous targeted metabolomic study it was shown that plasma ratios of alanine to branched-chained amino acids (BCAA) and of glycine to BCAA more accurately reflected duration and intensity of hypoxia in newborn piglets than the traditionally employed plasma lactate concentration [1].

The study of alterations of the metabolic profile within the central nervous system under hypoxic conditions (e.g. perinatal asphyxia) might yield complementary information to that provided by plasma and urine analysis thus leading to the identification of reliable biomarkers of brain injury. Retinal tissue is an integral neuronal tissue directly communicating and sharing many functional and structural characteristics with the brain [16]. Moreover, retina has a uniquely high metabolic demand for oxygen compensated by a highly efficient vascular supply [17] and it is considered one of the most oxygen-sensitive tissues. Hypoxia induces adaptive responses in the retina including changes in blood flow, protective metabolic adaptations, and angiogenesis [17].

The primary objective of this study was to assess metabolic changes that could be representative of the intensity and duration of hypoxia in an integral neuronal tissue. Facing the impossibility of analyzing retinal samples in human clinics, the present study was focused on metabolomic changes in the retina after an intense period of postnatal hypoxia and its comparison to control animals in room air in a well-established newborn piglet model of hypoxia, which reliably reflects pathophysiologic changes in the retina [1], [18]. Regardless of the analytical and biostatistical strategy followed, initial identification of differentiating metabolites in discovery studies based on the analysis of relatively reduced sample sets cannot be extrapolated to the whole population because there is merely not enough information to model the biological variability. Nonetheless, results are of great value in basic research in the hypothesis generation phase to guide further investigations eventually leading to a ‘biomarker qualification’ [19]. This study is aimed to guide further research for the development of highly reliable non-invasive biomarkers of brain injury secondary to birth asphyxia that could substantially improve clinical grading, therapeutic intervention strategy and prognosis.

## Materials and Methods

### Ethics Statement

The animal studies were carried out at Oslo University Hospital (Norway). The National Animal Research Authority (NARA) approved the experimental protocol. The animals were cared for and handled in accordance with the European Guidelines for use of Experimental Animals by certified FELASA fellows (Federation of European Laboratory Animals Science Association).

### Chemicals and Reagents

All the solvents were of ‘LC-MS’ grade and were purchased from Fisher Scientific (Loughborough, UK) or from Scharlau (Barcelona, Spain). Additives and standards were purchased from Sigma-Aldrich Quimica SA (Madrid, Spain). Commercially available pharmaceutical Somazina® 500 mg containing CDP-choline at a concentration of 125 mg/mL was purchased from Ferrer International (Barcelona, Spain).

### Animal Model and Sample Processing

A total of 10 newborn Noroc (LyxLD) piglets with the inclusion criteria of age between 12–36 h, hemoglobin >5 g/dL and good general conditions were selected for the study. The piglets were anaesthetized, orally intubated and surgically prepared as previously described [1]. Anesthesia was induced by giving sevofluran 5% (Sevorane, Abbott); an ear vein was cannulated, the piglets were given pentobarbital sodium 15 mg kg^−1^ and fentanyl 50 µg kg^−1^ intravenously as a bolus injection. The piglets were orally intubated then placed in the supine position and washed for sterile procedures. Anesthesia was maintained throughout the experiment by continuous infusion of fentanyl (50 µg kg^−1^ h^−1^) and midazolam (0.25 mg kg^−1^ h^−1^) in mixtures giving 1 mL kg^−1^ h^−1^ for each drug applied by IVAC P2000 infusion pump. When necessary, a bolus of fentanyl (10 µg kg^−1^), midazolam (1 mg kg^−1^) or pentobarbital (2.5 mg kg^−1^) was added (need for medication being defined as shivering, trigging on the respirator, increased tone assessed by passive movements of the limbs, increase in blood pressure and/or pulse). A continuous IV infusion (Salidex: saline 0.3% and glucose 3.5%, 10 mL kg^−1^ h^−1^) was given until hypoxia, or the corresponding time point for the Control group, and then 5 mL kg^−1^ h^−1^ throughout the experiment. The piglets were ventilated with a pressure-controlled ventilator (Babylog 8000+; Drägerwerk, Lübeck, Germany). Normoventilation (arterial carbon dioxide tension (PaCO_2_) 4.5–5.5 kPa) and a tidal volume of 6–8 mL kg^−1^ were achieved by adjusting the peak inspiratory pressure or ventilatory rate. Ventilatory rate was 15–40 respirations/min. Inspiratory time of 0.4 s and positive end-expiratory pressure of 4.5 cm H_2_O was kept constant throughout the experiment. Inspired fraction of O_2_ and end-tidal CO_2_ was monitored continuously (Datex Normocap Oxy; Datex, Helsinki, Finland). The left femoral artery was cannulated with polyethylene catheters (Porex PE-50, inner diameter 0.58 mm; Porex Ltd Hythe, Kent, UK). Mean arterial blood pressure (MABP) was measured continuously in the left femoral artery using BioPac systems MP150-CE. Rectal temperature was maintained between 38.5 and 39.5°C with a heating blanket and a radiant heating lamp. Throughout the whole experiment there was a continuous surveillance of blood pressure, saturation, pulse, temperature and blood gas measurements. After surgery, the piglets were placed in the prone position. One hour of stabilization was allowed after surgery.

#### Experimental Protocol

A total of 10 piglets underwent the experimental procedure. Out of these, 5 were randomly assigned to hypoxia (experimental group) and the remaining 5 to room air (control group). Hypoxemia was achieved by ventilation with a gas mixture of 8% O_2_ in N_2_ until either the mean arterial blood pressure decreased to 20 mmHg or the base excess (BE) reached −20 mmol/L. CO_2_ was added during hypoxemia aiming at a PaCO_2_ of 8–9.5 kPa to imitate perinatal asphyxia. At the end of hypoxia, or at the corresponding time point for the control group, the eyes were extracted (from the full anesthetized piglets), placed on an ice-cold glass plate and excised to quickly remove the retina of each eye. Once the retinas were obtained, they were frozen on liquid N_2_ and stored at −80°C. After the sampling of retinal tissue, the animals were given a lethal dose of pentobarbital (150 mg/kg iv). The left retinas were shipped on dry ice to the Perinatal Research Group of the *Health Research Institute Hospital La Fe* (Valencia, Spain) for processing and analysis. Samples were stored at −80°C until processed. Samples were homogenized in CH_3_OH:H_2_O (70∶30%v/v) using a PreCellys 24 dual Homogenizer (Bertin Tech., Montigny-le-Bretonneux, France) for a higher repeatability among replicates, employing a single pulse of 30 s and keeping samples at approx. 4^o^C using a Cryolys (Bertin Tech). Solvent volumes were adjusted to reach final concentrations of 33.3 mg frozen tissue/ml PBS buffer. After homogenization, samples were centrifuged for 10 minutes at 12000 rpm and 4°C and then supernatants were stored at −80°C until UPLC-QTOFMS and UPLC-MS/MS analysis.

### Metabolite Profiling

Metabolite profiling analysis of the retina extracts was performed in an Acquity UPLC-QTOF MS instrument (Waters, Milford, MA, USA). Sample extracts were analyzed by triplicate using an HSS T3 (100×2.1 mm, 1.8 µm, Waters) C18 column with a HSS T3 VanGuard precolumn (5×2.1 mm, 1.8 µm, Waters). 80 µL aliquot of the sample was placed in a clean Eppendorf tube and 9.6 µL of an aqueous solution containing 0.83% (v/v) HCOOH and 0.42 µg/mL reserpine (m/z 609.2812) as internal standard (IS) were added. Column and auto-sampler temperatures were set at 40°C and 4°C, respectively. A 15-min linear gradient elution was performed at a flow of 440 µL/min as follows: initial conditions of 100% of solvent A (H_2_O 0.1% v/v HCOOH) were kept for 0.5 min, followed by a linear gradient from 0% to 95% of mobile phase B (CH_3_OH) for 7.5 min; isocratic conditions of 95% B were held for 3.5 min; finally, a 0.5 min gradient was used to return to the initial conditions, which were maintained for 3 min. The eluting analytes were detected using a QTOF SYNAPT HDMS spectrometer (Waters, Milford, MA, USA). The following electrospray ionization (ESI) parameters were selected: capillary and cone voltages were set at 3.5 kV and 35 V in the positive mode; desolvation and source temperatures were set at 340°C and 120°C, respectively; flow rates of the cone and nebulization gases were set at 60 L/h and 800 L/h, respectively. Full scan data were collected in the TOF MS mode from 50 to 950 mass to charge ratio (m/z) with a scan time of 0.08 s. A Lock Spray interface (Waters) was used to maintain mass accuracy during the analysis. To this end, a 50 pg/mL solution of leucine enkephalin in CH_3_CN:H_2_O (1∶1) (0.1%v/v HCOOH) was infused every 10 s by using an isocratic pump at a flow rate of 40 µL/min, and its MS spectrum was acquired using a scan time of 0.2 s. The data station operating software used was MassLynx 4.1 (Waters). Sample acquisition was randomized to avoid the effect of potential instrument drifts during the sample batch measurement thus minimizing or averaging instrumental sources of variation that might bias the results. In addition, 7 blank extracts were analyzed for background correction and to monitor the lack of cross-contamination. Metabolite putative identification based on MS data was performed using both, the human metabolome database (HMDB, http://www.hmdb.ca) and the MassBank (MassBank, http://www.massbank.jp) open databases using a spectral mass tolerance of ±5 mDa. Identification of pyroglutamic acid (HMDB00267), CDP-choline (HMDB01413) and oxidized glutathione (GSSG) (HMDB03337) was confirmed by analysis of standard solutions under the same instrumental conditions.

### Quantitative Analysis of Choline, Acetylcholine and CDP-choline

Quantitative analysis of choline, acetylcholine and CDP-choline was performed using an Acquity UPLC system coupled to a Xevo-TQ triple quadrupole MS detector with an ESI (Waters, Manchester, UK) and a Kinetex™ (50×4.6 mm, 2.6 µm, 100 Å) HILIC column from Phenomenex (Torrance, CA, USA). A 6 min binary gradient elution at a flow rate of 400 µL/min was performed as follows: the mobile phase composition was maintained constant at 5% B (CH_3_OH) during 1.5 min, and then the gradient ran from 5% B to 5% A (H_2_O, 0.5% HCOOH) in 0.1 min and remained at 5% A for 1 min before it was returned to 5% B in 0.1 min for equilibration during 3.5 min. Positive ESI and detection conditions were as follows: capillary voltage was set to 3.5 kV, cone voltage to 10 V, source temperature to 120°C and the cone, desolvation and collision gas flows were set to 50, 700 and 0.2 L/h, respectively.

Stock standard solutions were prepared in water by direct weighing. A set of 10 standard solutions was prepared by serial dilution in water in the concentration ranges summarized in Table 1. Tandem MS (MS/MS) detection was used for quantification using the acquisition parameters summarized in Table 1. Ionization and fragmentation parameters were optimized, for all analytes under study, by analyzing separate standard solutions at a concentration of 50 µM. The analysis of individual standards confirmed the lack of spectral interferences among the selected analytes. Linear external calibration lines were obtained from UPLC-MS/MS peak area measurements. Confirmation of the identity of the acetylcholine and CDP-choline in samples was based on the following criteria: i) both acquired Multiple Reaction Monitoring (MRM) transitions must occur at the same time; ii) the relative abundance of the MRM signals must be within ±25% of the one observed in a standard with a similar concentration; and iii) the MRM transitions must have signal to noise ratios higher than 9. Identification of choline was based on the retention time due to the lack of appropriate additional MS fragments.

**Table 1:** Main MS acquisition parameters employed for MRM.

Metabolite	RT (min)	Quantification MRM	Confirmation MRM	CE [eV]	Cone [V]	R^2^	SNR^a^	Linear range [nM]
Choline	2.33±0.01	104.1>60.2	-	15	40	0.96	35	19.5–10000
Acetylcholine	3.95±0.02	146.2>87.1	146.2>60.1	15	40	0.997	451	19.5–2500
CDP-choline	2.532±0.008	489.1>264.1	489.1>360.1	20	35	0.9998	19	19.5–10000

Note: SNR stands for signal-to-noise ratio and was calculated from data obtained during the analysis (n = 3) of a standard solution at a concentration of 19.5 nM.

### Data Analysis

Raw MS data was processed using MarkerLynx XS V4.1 (Waters, Milford, MA, USA) and the following main parameters: peak baseline noise: 6 a.u.; peak width at 5% height: 8 s; marker intensity threshold: 10 counts; retention time window: 0.15 min; mass window: 0.02 Da, and activated deisotopic filter to remove C^13^ signals from the markers table. Peak detection, integration and alignment were carried out across all samples and blank injections providing a raw data matrix X_0_ (37×365) with samples in rows and their corresponding features (i.e. variables) in columns. First, the median values of each variable calculated from the within-sample replicate measurements (n = 3) of the retina extracts were calculated, thus obtaining an X_1_ (10×365) data matrix. Then a data set X_2_ (10×229) was obtained after removal of a total of 136 background variables detected in blank injections, likely arising from sample collection, storage and background contamination. Finally, variables present in less than 3 retina samples in the data set were eliminated, thus obtaining the data matrix X_3_ (10×112). The mean number of variables detected for normoxic (96±4) and hypoxic (97±4) samples in X_3_ were found to be statistically comparable. Preprocessing, normalization and multivariate analysis of UPLC-QTOFMS data was run under Matlab 7.7.0 (Mathworks Inc. Natick, MA, USA) using in-house written Matlab functions and the PLS Toolbox 7.0 from Eigenvector Research Inc. (Wenatchee, USA). Raw data and Matlab files are available from the authors or at the website www.perinox.es.

#### Principal component analysis (PCA) and hierarchical cluster analysis (HCA)

A PCA model of the X_3_ (10 x 112) metabolomic data set was built using autoscaling as data pretreatment. Here, the number of Principal Components (PCs) was selected by leave-one-out cross validation (LOO-CV). The residual Q and the Hotelling’s T^2^ statistics were calculated for outlier detection: the Q-statistic represents the Euclidean distance between a sample and its projection onto the multivariate model. For those samples whose Q value is outside the 95% confidence interval, the model would be not valid due to the existence of a new source of variation absent from the calibration dataset. Hotelling’s T^2^, the sum of normalized squared scores, measures the variation of each sample within the model.

Agglomerative hierarchical cluster analysis (HCA) was employed for initial unsupervised exploratory data analysis using the Ward’s method and Euclidean distances to determine the distances between samples in the PCA model. This method joins the two existing clusters such that the resulting pooled within-cluster variance with respect to the centroid of each cluster is minimized.

#### Partial least squares - discriminant analysis (PLSDA)

Supervised PLSDA was performed employing the nonlinear iterative partial least squares (NIPALS) algorithm, a maximum number of latent variables of 3, and autoscaling as data pretreatment. The y vector containing the class labels (i.e. 0 or 1 for hypoxia and normoxia samples, respectively) was mean centered. The residual Q and the Hotelling’s T^2^ statistics were calculated for outlier detection.

In order to avoid model overfitting, PLSDA figures of merit were calculated by means of leave-one-out double cross validation (2 CV). The selection of the inner-PLS model dimensionality was based on discriminant Q^2^ [20] calculated by leave-one-out cross validation. The statistical significance of the PLSDA model was assessed by a non-parametric permutation test in which the null distributions of the figures of merit were estimated by randomly permuting the class labels of the samples, as described elsewhere [21]. In this work, the permutation test included all possible non-complementary combinations taking 5 elements of each class at a time for PLSDA modeling.

#### Selection of differentiating metabolites

The selection of differentiating metabolites was carried out using the distribution of mean PLS regression vectors obtained during the permutation test (b_random_) and the mean regression vector calculated using real class labels (b_real_): for each variable, a p-value was computed as the fraction of permuted statistics that are at least as extreme as the test statistic obtained using real class labels [21]. Those variables whose p-value was <0.025 where classified as differentiating metabolites. A detailed description of the algorithm can be found elsewhere [21], [22], [23].

## Results

### Cohort Characterization during the Experiment

The summary of the cohort characteristics measured after 1 h stabilization/relaxation procedure before and after the intervention is given in Table 2. Clinical parameters including mean arterial blood pressure (MABP), base excess (BE), temperature, pH, pCO_2_, pO_2_ and lactate measured after a one hour stabilization/relaxation procedure did not differ between the experimental (i.e. hypoxia) and control (i.e. normoxia) groups (*p*-values>0.05) before intervention. The time of hypoxia needed to reach a decrease in mean arterial blood pressure to 20 mmHg or the base excess (BE) to reach −20 mmol/L ranged from 45–85 minutes.

**Table 2:** Clinical parameters of the study cohort measured after a one hour stabilization/relaxation procedure before and after intervention.

	Before intervention			After intervention		
	Normoxia	Hypoxia	p-value	Normoxia	Hypoxia	p-value
**MABP (mmHg)**	63(±137)	54 (±20)	0.4	66 (±19)	18.6 (±2)	0.001
**BE (mmol/L)**	2.5(±1.8)	5 (±3)	0.15	3.6 (±1)	−19 (±3)	<0.001
**Temperature**	38.9 (±0.9)	39.5 (±0.4)	0.25	39.1 (±0.6)	38.8 (±0.2)	0.37
**pH**	7.44 (±0.07)	7.45 (±0.05)	0.48	7.44 (±0.03)	6.9 (±0.1)	<0.001
**pCO_2_ (kPa)**	5.2 (±0.9)	5.5 (±0.8)	0.61	5.5 (±0.4)	9.5(±1)	<0.001
**pO_2_ (kPa)**	11 (±1.3)	11.6 (±1.3)	0.85	11 (±1.3)	5.0 (±0.6)	<0.001
**Lactate (mmol/L)**	2.5 (±0.9)	1.3 (±0.4)	0.33	1.5 (±0.6)	12.9 (±3.7)	0.002

Values are presented as mean (± standard deviation). p-values correspond to two-groups comparisons using Student’s t-test for independent data.

### Metabolomic Analysis of Retina Tissues

#### UPLC-QTOFMS data quality

Figure S1 depicts instrument performance parameters monitored throughout the sample batch measurement: peak area (Figure S1–A) and mass accuracy (Figure S1–B). Stability of the overall instrument sensitivity, estimated as the relative standard deviation (RSD) of the internal standard peak area through the sample batch, was equal to 14%. Mass accuracy, measured as the relative error (ppm) of the molecular ion of the internal standard at the peak apex, ranged ±10 ppm through the sample batch. Figure S2 shows histograms of the %RSD values calculated from the replicate analysis of the retina sample before (A) and after identification of the set of differentiating metabolites (B). Figure S2–C depicts scatter plots showing the correlation found among replicates of a typical retina extract.

#### Principal component analysis (PCA) and hierarchical cluster analysis (HCA)

**Figure 1:**
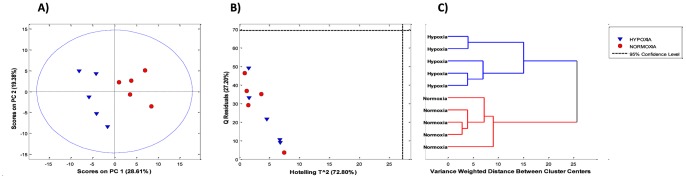
Principal component analysis of the metabolomic profiles of retina tissues. A) Score plot of PC1 vs. PC2; B) residual Q versus the Hotelling’s T^2^ statistics; C) dendrogram obained by hierarchical cluster analysis.

A PCA model was initially built using 4 PCs explaining together 72.8% of the total variance of the original data set. In the obtained PC1–PC2 scores plot depicted in Figure 1A, a remarkable separation of the samples into hypoxia and normoxia groups was observed. No sample was classified as outlier, as none of the samples fell outside the depicted 95% confidence level calculated assuming a normal scores distribution (see Figure 1A). Projections in later components did not provide additional sample clustering (results not shown). Besides, calculated Q and Hotelling’s T^2^ statistics confirmed that all samples fall within the 95% confidence levels (see Figure 1B). Figure 1C shows the obtained dendrogram after PCA of the data confirming the expected clustering between hypoxia and normoxia samples.

#### Partial least squares discriminant analysis (PLSDA)

**Figure 2:**
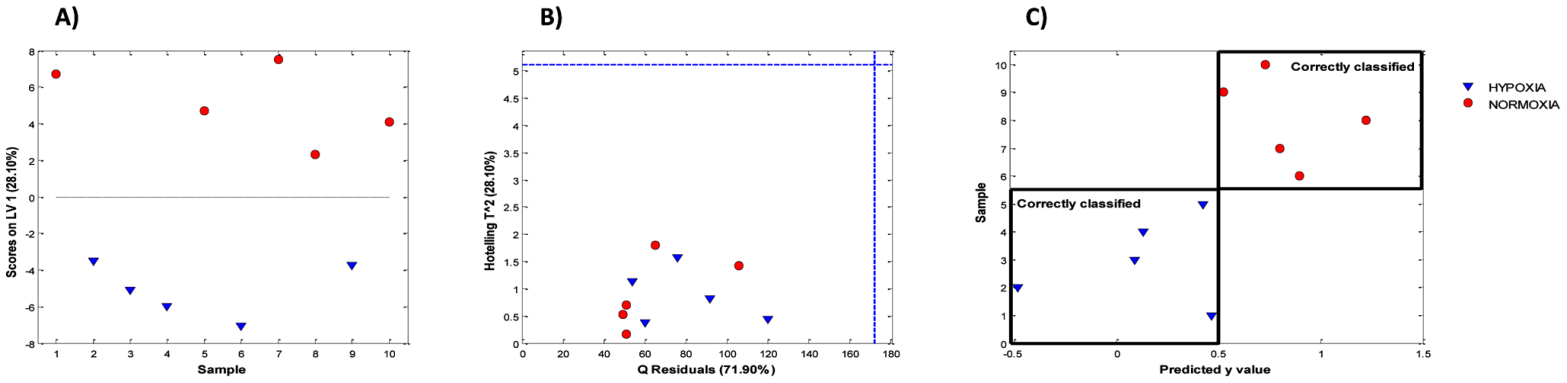
Partial least squares - discriminant analysis of the retina metabolome between normoxic and severely hypoxic newborn piglets. A) PLSDA scores plot; B) residual Q versus the Hotelling’s T^2^ statistics; C) PLSDA predicted class labels by double cross validation.

Initially, a supervised PLS model was calculated to discriminate between hypoxic and normoxic samples. The number of latent variables (LV) of this model (LV = 1) was selected from dQ^2^ values calculated by LOO-CV. No subgroup could be identified from the PLSDA scores plot shown in Figure 2A, in good agreement with results obtained by PCA (see Figure 1A). The residual Q and the Hotelling’s T^2^ statistics depicted in Figure 2B showed that all the samples were within the 95% confidence intervals of both statistics.

Figure 2C shows the PLSDA predicted *y* values calculated by 2CV. From these values, two figures of merit were calculated: dQ^2^ and the number of misclassified samples (NMC) which provided dQ^2^ = 0.69 and NMC = 0. Statistical validation of both figures of merit was performed by means of a non-parametric permutation test as described before obtaining empirical *p*-values<0.01 for both statistics.

#### Selection of differentiating metabolites

The set of PLS regression vectors obtained during the permutation test was used to identify a total of 8 variables as differentiating metabolites (*p*-value<0.025) (see Figure S3). Results obtained from the identification of the selected variables based on their *m/z* values are summarized in Table 3. As previously stated, *m/z* based identifications of CDP-choline, pyroglutamic acid and GSSG from TOFMS data were further confirmed by analyzing available reference standard solutions, leaving two of the selected signals unidentified.

**Table 3:** List of selected differentiating metabolites from the retina data set.

Nr.	Retention time[min]	m/z	Metabolite
**1**	0.57	84.0451	Pyroglutamic acid^(1)^
**2**	0.57	130.0506	Pyroglutamic acid
**3**	0.53	201.9340	Unidentified
**4**	0.54	222.0302	Unidentified
**5**	0.53	364.9069	CDP-DG^(2)^
**6**	0.69	489.1157	CDP-choline
**7**	0.69	511.0977	CDP-choline^(2)^
**8**	0.77	613.1588	GSSG

(1): Pyroglutamic in-source fragment [M+H-CH_2_O_2_]^+^; (2): Na adduct.

**Figure 3:**
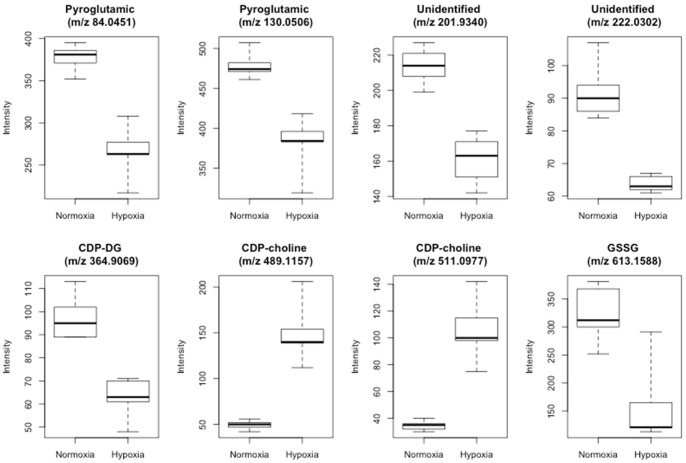
Intensities of the selected differentiating metabolites. Boxplots showing UPLC-QTOF intensities of the selected discriminant metabolites in retina of normoxic and severely hypoxic newborn piglets.

**Figure 4:**
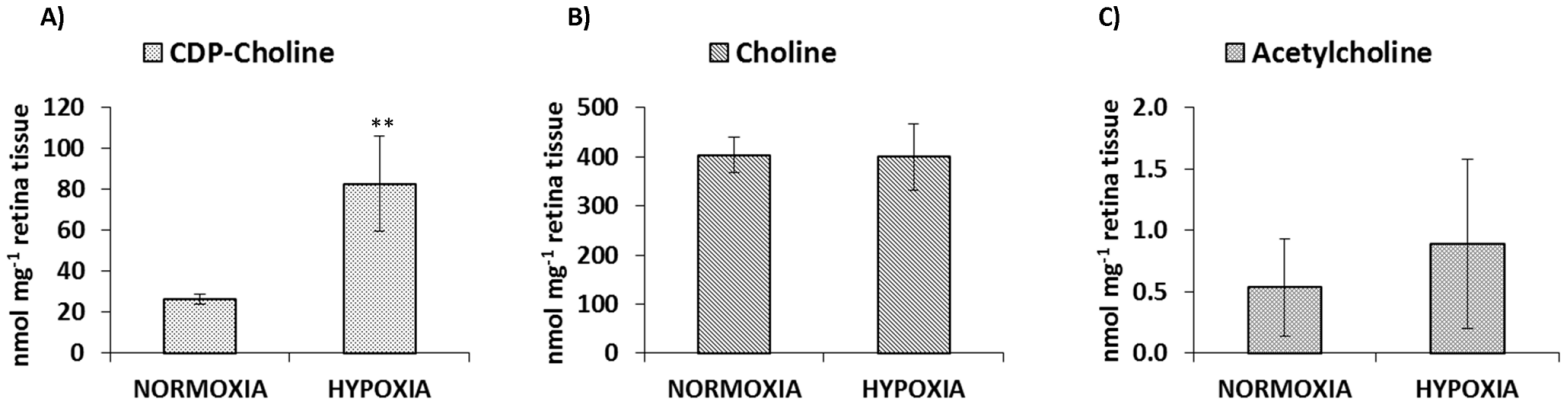
Concentrations of choline, acetylcholine and CDP-choline found by target analysis. Mean concentrations of CDP-choline (A) choline (B), acetylcholine (C) and (expressed as nmol/mg of tissue) in retina samples as determined by UPLC-triple quadrupole MS/MS.

Figure 3 shows boxplots of the UPLC-QTOFMS intensities of the differentiating metabolites. Results showed statistically significant differences between levels for normoxic and severely hypoxic piglets (t-test, p-value<0.005). In order to confirm results obtained by the untargeted approach a quantitative HILIC-UPLC-MS/MS method for CDP-choline, choline and acetylcholine in retina samples was developed. Quantification of CDP-DG could not be included in the present study due to the lack of commercially available standards. Typical chromatograms are shown in Figure S4. Results summarized in Figure 4A confirmed an increase in CDP-choline levels under hypoxic conditions (*p*-value<0.01). On the contrary, no statistically significant difference was found between acetylcholine and choline levels under hypoxia and normoxia (see Figure 4, B and C).

## Discussion

### Metabolomic Data Quality and Pre-processing

Due to the wide detection capabilities of UPLC-QTOFMS, minor differences in sample composition and/or measurement conditions give rise to high numbers of missing values, that can originate from different sample compositions (e.g. metabolites that are absent or below the limit of detection in sample subsets) or from faulty peak alignments. Features with a low frequency of detection are not likely to be reliable markers. They reduce the robustness and predictive capabilities of multivariate models [24] and might hamper a further biochemical interpretation of the results. Initial unsupervised data pre-processing described in section *Data analysis* allowed the elimination of 253 variables (69% of the total), reducing the variable-to-sample ratio from 36.5 to 11.2. Besides, it also provided statistically comparable numbers of variables detected for normoxic (96±4) and hypoxic (97±4) samples, and reduced the number of missing values in the data set from 33% to 14% of the total. In summary, the initial variable elimination reduced the probability of finding chance correlations.

This study focused at finding biologically relevant differences in the retina metabolome of newborn piglets (12–36 h) subjected or not to hypoxia. Therefore, it was of importance to assess that the observed differences were not caused by instrumental sources of data variation such as solvent contaminants, cross-contamination or sensitivity drifts. Results depicted in Figure S1 (i.e. overall instrument sensitivity equal to 14% and mass accuracy in the ±10 ppm range) confirmed the stability and suitability of the instrument performance for obtaining reliable metabolomic profiles.

Variability among sample replicates is very important to accept or reject data as it might be indicative of a low instrumental stability or sample degradation during time-consuming sample measurement and hampers the identification of minor changes due to hypoxia. In this work, variability among replicates was evaluated by both, their RSD% of the detected signals and the correlation coefficients among the metabolomic profiles. Figure S2 depicts the histograms of the RSD% values using the 112 retained variables (A) and a selected set of discriminant variables (B), where it can be seen that the mean RSD% calculated for the selected discriminant variables was comparable to that found for the internal standard (14%). Besides, high correlation coefficients (R>0.95) among the replicates were found (see Figure S2–C).

In summary, results confirmed the quality of the data as the stability of the sensitivity and mass accuracy levels was ensured, and the precision of the sample replicate measurements provided results comparable to those found for the internal standard.

### Sample Size Evaluation and Exploratory Data Analysis

Selection of the sample size required to derive a statistically meaningful estimation in metabolomics is critical to produce robust results. Nonetheless, there is no single criterion to estimate it in advance for highly collinear multivariate data sets typically found in metabolomics studies. In this study, the required sample size depended on a number of *a priori* unknown factors including the size of the effect of hypoxia and its variation among the individuals, the pre- and post-intervention biological variation of the detected metabolites, as well as the analytical variation (e.g. during sample collection, storage, pre-treatment and instrumental measurement). Besides, the number of animals included in the study should be kept to a minimum from an ethical standpoint [25] and because of that both, overpowered and underpowered studies are undesirable and a compromise must be ensured [26]. The group size is often small in piglet studies [27] [28] [29] [30], sometimes even having group sizes limited down to 2–5 piglets in each group [31]. Therefore, two aspects were taken into account for sample size selection: i) the cohort of piglets formed a homogeneous group due to their high environmental and physiological similarity (see Table 2), and ii) it was reasonable to expect a high size effect of hypoxia on the retina metabolome due to its extreme physiological effects on the piglets. Besides, the retina is rich in polyunsaturated fatty acids, which might be at risk of being oxidized by reactive nitrogen and oxygen species [32] [33].

Sample size selection criterion was supported by results obtained from the explorative analysis of the data set. PCA is one of the most commonly used methods for exploratory data analysis. It provides an unbiased overview of the data structure and it is a common strategy for the estimation of whether outliers, trends or groups are present in multivariate data sets. The PCA scores plot provides a direct illustration of the latent patterns in the data set as the distance between objects (i.e. samples) is related to their similarity with respect to what patterns the model describes [34]. From the scores plot shown in Figure 1A, it was apparent that the effect of hypoxia dominated PC1 variation which describes 28.6% of the total variance of the data, and no within-class clustering could be observed (see Figures 1A–B). This was confirmed by the dendrogram calculated by HCA depicted in Figure 1C. Although the number of groups in the dendrogram depends on the choice of the distance cutoff, it was clear that the high size of the effect of hypoxia on the retina metabolome split the data into two clusters (i.e. normoxia and hypoxia samples). Therefore both, the size of the effect and the within-class homogeneity of the retina samples supported the use of the selected sample size to provide adequate power to the analysis. This limited-size study aimed at providing a characterization of the metabolomic response of retinal tissue caused by profound hypoxia in a well-established neonatal piglet model for the identification of candidates of hypoxia biomarkers. However, the study was essentially designed for hyptohesis-generation and the usefulness, performance and biochemical significance of the candidate biomarkers must be validated in larger studies.

### Supervised Discriminant Analysis

Supervised PLSDA provided a better interpretation of the effect of hypoxia on the retina metabolome, allowing the assessment of the class separation and the identification of a subset of discriminant metabolites. Initially, a PLSDA model was calculated. The use of a single latent variable was chosen from dQ^2^ values calculated by leave-one out cross validation. The PLSDA scores plot shown in Figure 2A did not reveal subgroups within the hypoxia or normoxia groups of samples. Moreover, from the residual Q and the Hotelling’s T^2^ statistics depicted in Figure 2B, no sample was classified as outlier. Both results were in good agreement with the results obtained by PCA.

When variables outnumber samples, false discovery rates and premature claims of significance of PLSDA models due to, for example random chance correlations and model over fitting represent a major problem [22], [35] and model validation becomes mandatory. The gold standard for validation is the use of a representative external data set not used for model development [36], [37]. Nonetheless, cross model validation (2CV) has been repeatedly employed [21], [22], [23] as a suitable alternative in metabolomic studies when no external set is available that provides external figures of merit. In leave-one-out 2CV one of objects is set aside as a test set. The remaining set of objects are again split into training and validation sets and subjected to a leave-one-out standard cross validation for the selection of the number of latent variables [38].

Results from 2CV of the PLSDA model are depicted in Figure 2C. Results showed that all the samples were correctly classified (NMC = 0) also providing dQ^2^ = 0.69. The statistical significance of NMC and dQ^2^ figures were established using a non-parametric permutation test providing *p*-values<0.01 for both statistics, thus supporting the existence of a significant effect on the retina metabolome caused by hypoxia which could not be attributed to chance or model overfitting.

The compounds selected summarized in Table 3 were considered as discriminant metabolites and thus classified as potential biomarkers of hypoxia. To obtain further information on the results, univariate plots of the intensities of these metabolites were depicted indicating clear up or down regulations of the metabolites (see Figure 3). Although multivariate and univariate plots cannot be directly compared, results found facilitated the biological interpretation of the results. Besides, the use of the newborn piglet model enables us to exactly know the time of hypoxia and we could thereby correlate the selected set biomarkers to the duration of hypoxia. The possibility of prediction of the duration of hypoxia by quantification of endogenous plasma metabolites (i.e. ratios of Ala/BCAA, Gly/BCAA, succinate and propionyl-L-carnitine) has been recently reported [1] opening a new field of research. In spite of the observed differences on the levels of the selected metabolites between both groups further research is needed to assess their correlation with the duration of hypoxia. Quantitative analysis of CDP-choline concentration in the retina extracts by HILIC-MS/MS confirmed results obtained by UPLC-QTOFMS (see Figures 3 and 4).

### Biological Interpretation

When selecting signals as potential biomarkers, their discriminant capability and physicochemical properties as well as the biochemical relation with the topic of investigation were considered. GSSG (oxidized glutathione) was identified as a differentiating metabolite and in fact, the ratio of reduced glutathione (GSH) and GSSG is typically employed as an indicator of the oxidative status of biological systems. However, accurate quantification of GSH in biological matrices requires a previous derivatization step using, e.g. N-ethyl-maleimide [39], [40] to block free sulfhydryl groups thus avoiding oxidation into GSSG. Therefore, as the acquired signal might be biased, GSSG was not classified as a potential biomarker in this data set. Nonetheless, quantitative analysis of GSH/GSSG using a dedicated analytical protocol will be the subject of future studies.

Analogous, pyroglutamic acid (also known as 5-oxoproline), which was also identified as differentiating metabolites, is involved in glutamate availability for GSH synthesis and its accumulation induces oxidative stress [41]. Under hypoxic conditions, GSH synthesis is activated to compensate the high GSH consumption by the retina [42]. Of note, re-cycling of glutamate, a relevant neurotransmitter, from the synaptic space during hypoxia is blunted due to the lack of ATP causing its accumulation in the neuronal tissue. However, its concentration rapidly decreases upon reoxygenation and therefore cannot be considered a reliable biomarker for the intensity of hypoxia [43].

CDP-choline is the limiting intermediate compound in the major pathway of the phosphatidyl-choline biosynthesis (i.e. the Kennedy pathway) [44]. Together with its hydrolysis products, namely cytidine and choline, CDP-choline plays an important role in the generation of phospholipids involved in membrane formation and repair. Moreover, it contributes to important metabolic functions such as formation of nucleic acids, proteins and acetylcholine [45]. Neuroprotective properties of endogenous CDP-choline have been known over two decades [3] and exogenously administered it prevents, reduces or even reverses effects of ischemia and/or hypoxia in different animal and cellular models. However, information regarding the role of endogenous CDP-choline is still scarce [3], [45]. It is known that during CDP-choline synthesis pyrophosphate, which is involved in energy transfer reactions such as glycolysis under anaerobic conditions, is released [44], [46]. Under normoxic conditions, as a result of the interaction of CDP-choline with diacyl-glycerol, phosphatidyl-choline and monoglycerides are produced. However, under hypoxic conditions, the normal reaction is reversed due to an increase in monoglycerides, and diacyl-glycerol is rapidly degraded to free fatty acids [44]. This is in agreement with results obtained in this study where elevated levels of CDP-choline and a decrease in CDP-DG were observed under hypoxic conditions (see Figure 3). Besides, previous works showed that induced apoptosis in HL-60 cells caused an increment of CDP-choline concentration suggesting an inhibition of phosphatidylcholine synthesis through inhibition of the enzyme CDP-choline:1,2-diacylglycerol cholinephosphotransferase (CPT, EC 2.7.8.2) [47]. CPT has an optimum pH range of 8.0–8.5 [48] and is inhibited by Ca^+2^ [45], [46]. Hypoxic insults to the brain modify nuclear membrane Ca^+2^ influx mechanisms (e.g. nuclear membrane IP(3) receptor) leading to increased intracellular levels of Ca^+2^ [48]. Results found in this work might also support the hypothesis of CDP-choline accumulation via CPT inhibition. However, further research is required to confirm this hypothesis. It is remarkable that under hypoxic conditions a rise in CDP-choline was observed whereas the other selected metabolites showed higher concentrations under normoxia. From results depicted in Figures 3 and 4, CDP-choline was classified as a promising biomarker for hypoxia in retinal (neuronal-like) tissue. However, validity of CDP-choline has yet to be confirmed upon resuscitation with further experiments in hypoxic animals resuscitated with different oxygen concentrations. Moreover, applicability of CDP-choline in the clinical setting and especially in the newborn period would request confirmation of its reliability in non-invasively attainable biofluids such as urine. This would allow serial measurements without performing invasive interventions.

### Conclusions

The untargeted metabolomic analysis of retina samples obtained from asphyxiated and control newborn piglets allowed the selection of eight differentiating metabolites. Results were statistically validated confirming their significance (*p*-value<0.01). Further validation of the results was conducted by targeted UPLC-MS/MS for the quantification of one of the identified differentiating metabolites (CDP-choline) and two related metabolites (choline and acetylcholine) confirming the obtained results.

Endogenous CDP-choline could be a promising candidate biomarker as its key role in phospholipid synthesis as well as neuroprotective function under hypoxic conditions are well known and even therapeutic effects are described extensively in literature. Future studies in larger populations and additional matrices (e.g. blood, serum or plasma, or urine) are going to be carried out in order to test the performance of CDP-choline as a biomarker for hypoxia in retina and its reliability upon resuscitation.

## Supporting Information

Figure S1 **Instrumental stability during the sample batch measurement.** A) Mass accuracy given as m/z error in ppm reserpine, lines: mean value +/− standard deviation; B) peak area values showed a 13.8% relative standard deviation (RSD).
(TIF)

Figure S2 **Repeatability among sample replicates.** A) Histogram of the %RSD values calculated from the replicate analysis of the retina samples for the set of 112 retained variables; B) Histogram of the %RSD values calculated from the replicate analysis of the retina samples using the set of differentiating metabolites; C) Scatter plot showing the typical correlation found among replicates of a retina extract. Dotted black line: linear regression line. Red solid line: theoretical 1∶1 diagonal line.
(TIF)

Figure S3 **Selection of differentiating variables.** Mean regression PLSDA vector obtained from leave one out 2CV and confidence boundaries calculated during the permutation test for the identification of discriminant metabolites (solid green circles).
(TIF)

Figure S4 **HILIC-UPLC-MS/MS typical chromatograms of choline, acetylcholine and CDP-choline.** Chromatograms of choline (A), acetylcholine (B) and CDP-choline (C) obtained from the injection of a standard solution (concentrations were 10 µM, 78 nM and 5 µM, respectively) and hypoxic and normoxic retina samples. Note: chromatographic conditions described in section *Quantitative analysis of choline, acetylcholine and CDP-choline.*
(TIF)
