# Supplementary figures and images for: Metabolomic Analysis of the Effect of Postnatal Hypoxia on the Retina in a Newly Born Piglet Model
Source: PLoS One. 2013 Jun 18;8(6):e66540. doi: 10.1371/journal.pone.0066540 (PMC3688918; doi:10.1371/journal.pone.0066540)

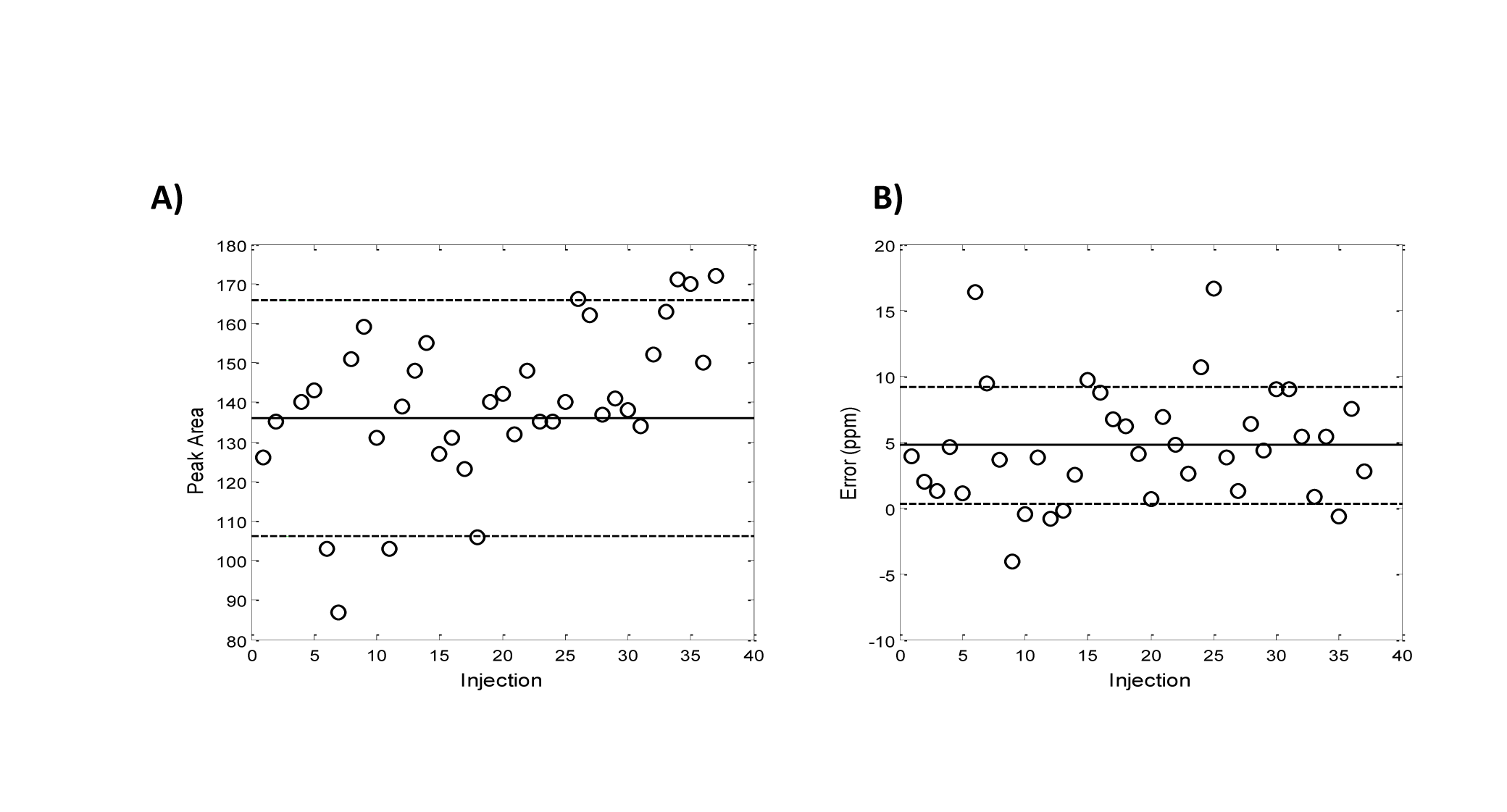

Supplement: Figure S1 — Instrumental stability during the sample batch measurement. A) Mass accuracy given as m/z error in ppm reserpine, lines: mean value +/− standard deviation; B) peak area values showed a 13.8% relative standard deviation (RSD). (TIF) [file pone.0066540.s001.tif]

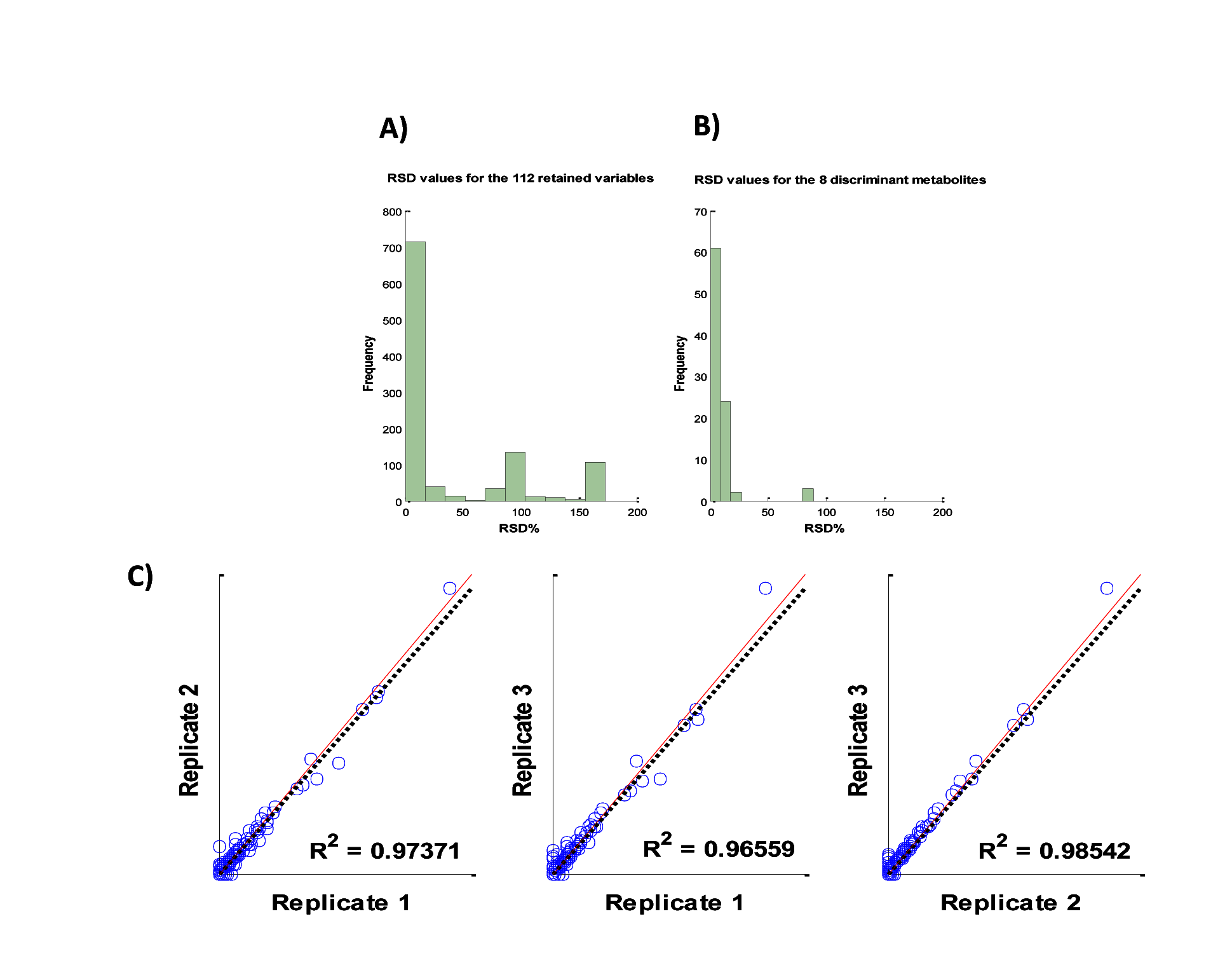

Supplement: Figure S2 — Repeatability among sample replicates. A) Histogram of the %RSD values calculated from the replicate analysis of the retina samples for the set of 112 retained variables; B) Histogram of the %RSD values calculated from the replicate analysis of the retina samples using the set of differentiating metabolites; C) Scatter plot showing the typical correlation found among replicates of a retina extract. Dotted black line: linear regression line. Red solid line: theoretical 1∶1 diagonal line. (TIF) [file pone.0066540.s002.tif]

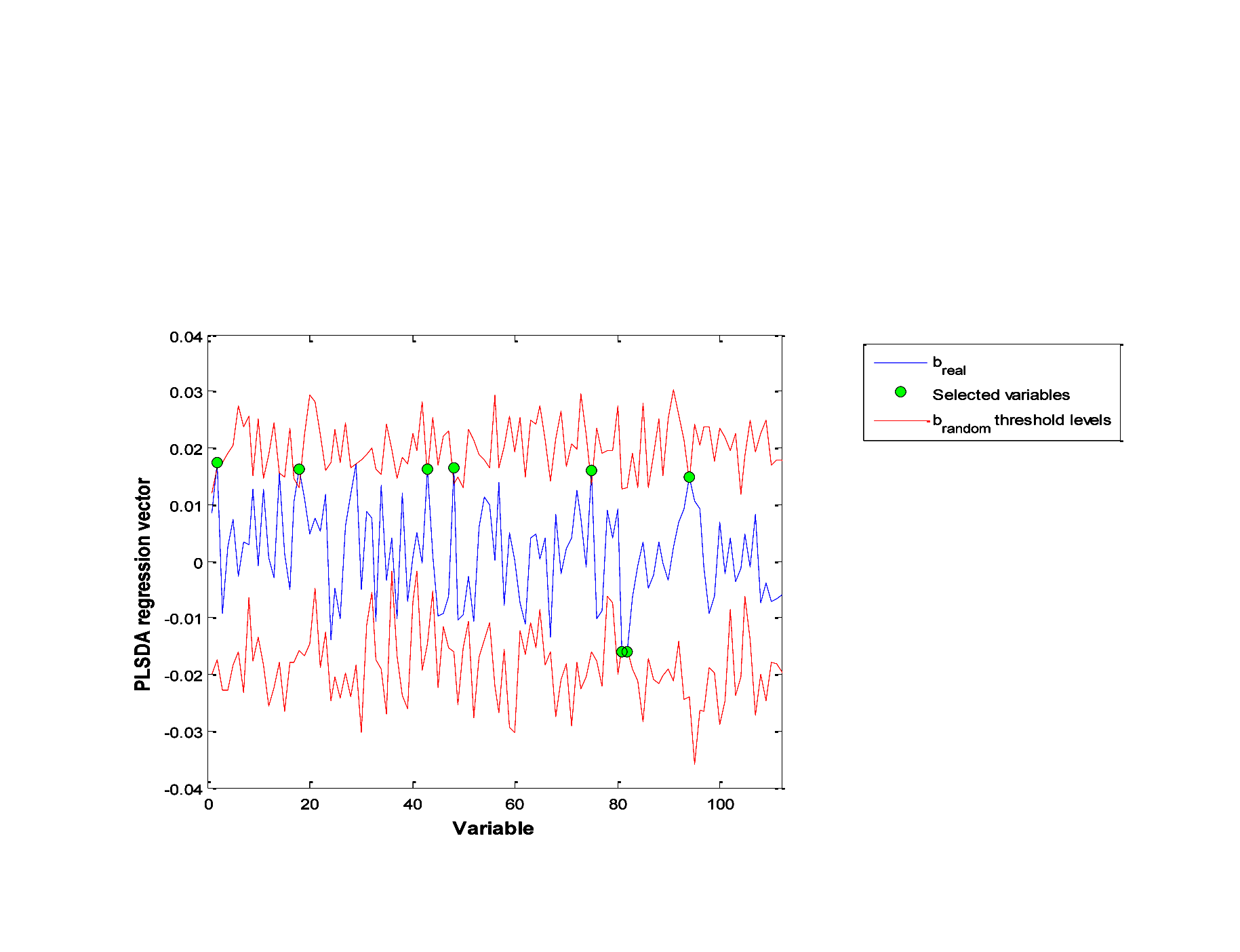

Supplement: Figure S3 — Selection of differentiating variables. Mean regression PLSDA vector obtained from leave one out 2CV and confidence boundaries calculated during the permutation test for the identification of discriminant metabolites (solid green circles). (TIF) [file pone.0066540.s003.tif]

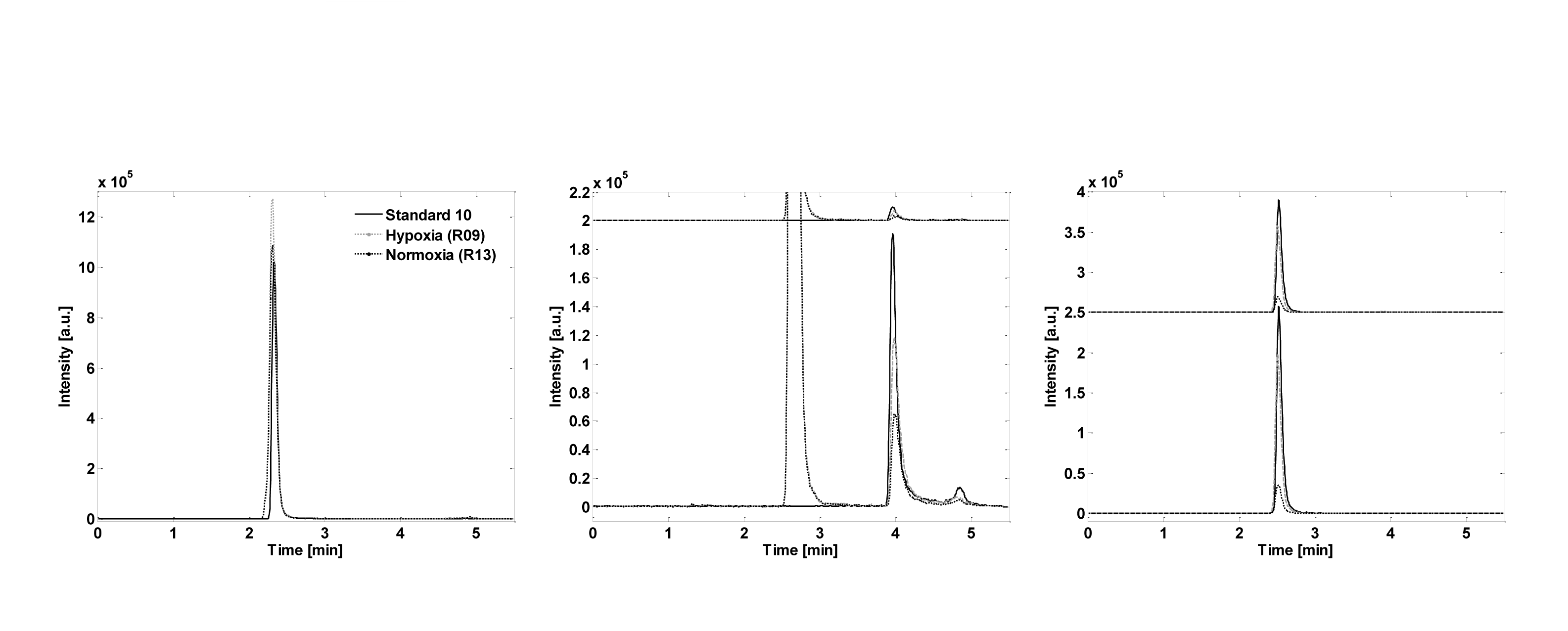

Supplement: Figure S4 — HILIC-UPLC-MS/MS typical chromatograms of choline, acetylcholine and CDP-choline. Chromatograms of choline (A), acetylcholine (B) and CDP-choline (C) obtained from the injection of a standard solution (concentrations were 10 µM, 78 nM and 5 µM, respectively) and hypoxic and normoxic retina samples. Note: chromatographic conditions described in section Quantitative analysis of choline, acetylcholine and CDP-choline. (TIF) [file pone.0066540.s004.tif]
